# Implementation of a Robotic Surgical Program With the Dexter Robotic Surgery System: Initial Experiences in Cholecystectomy

**DOI:** 10.1002/wjs.12531

**Published:** 2025-03-23

**Authors:** Anne‐Sophie Hotz, Nico Seeger, Lukas Gantner, Felix Grieder, Stefan Breitenstein

**Affiliations:** ^1^ Department of Visceral and Thoracic Surgery Cantonal Hospital Winterthur Winterthur Switzerland

**Keywords:** cholecystectomy, Dexter Robotic Surgery system, minimally invasive surgery, robot‐assisted surgery

## Abstract

**Background:**

The use of surgical robots in minimally invasive visceral surgery is increasing, with new platforms like the Dexter Robotic System. This study evaluated the implementation of Dexter in a general visceral surgery department, focusing on safety, performance, and surgeon stress in elective cholecystectomy.

**Materials and Methods:**

Three surgeons with varying laparoscopic and robotic experience performed robotic cholecystectomies with Dexter between December 2022 and June 2024. Perioperative outcomes and safety data were collected until 30 days post‐surgery. Surgeons' stress load and physical discomfort were assessed using validated questionnaires (SMEQ, STAI, and LED).

**Results:**

Fifty‐nine patients underwent elective gallbladder removal. Median age was 52 years (range 27–85) and BMI 26.3 kg/m^2^ (range 18.3–41.2). All surgeries were completed robotically without conversion to open surgery. There were no intraoperative complications or device deficiencies. Two cases were converted to laparoscopy due to patient anatomy and a liver tumor discovery. One postoperative complication (Clavien–Dindo grade 3A) involved choledocholithiasis requiring ERCP. Median total operating time, docking time, and console use time were 60 min (IQR 50–78), 5 min (IQR 4–7), and 23 min (IQR 19–34), respectively. Operative times revealed a fast‐learning experience, stabilizing after 10–15 cases. Surgeons reported high comfort (LED Median 3, IQR 0–6) and low stress (SMEQ median 10, IQR 10–26.25).

**Conclusion:**

The Dexter system was safely implemented in clinical practice, with efficient learning curve and low perceived stress, even for surgeons without prior robotic experience. Further studies are needed to determine whether Dexter offers advantages over conventional techniques.

## Graphical abstract

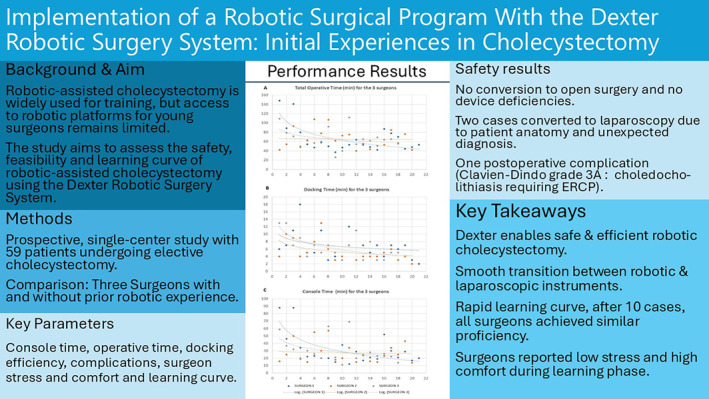

## 1 Introduction

Over the past few decades, minimally invasive techniques have become standard for many surgical procedures, offering benefits such as less postoperative pain, reduced blood loss, smaller incisions, and fewer complications [1, 2]. Robotic‐assisted surgery, now established in many specialties, provides advantages like wristed instrument mobility, improved ergonomics, tremor reduction, and a stable magnified visual field. The first robotic‐assisted cholecystectomy (RAC) was performed in 1997 [3] and has since been widely adopted [4, 5, 6]. Cholecystectomy is often used as a training operation to familiarize surgeons with robotic platforms and prepare them for more complex procedures [7, 8, 9]. Surgeons reported increased comfort and reduced physical and mental stress during RAC compared to laparoscopic cholecystectomy (LC) [10, 11].

Barriers to broader robotic adoption include high costs and the steep learning curve of implementing a robotic program [9, 12, 13]. The RAC learning curve previously reported ranged between 16 and 32 cases [14]. Robotic surgery also requires dedicated operating rooms (ORs), posing logistical challenges, and workflow inefficiencies due to OR size [15]. Additionally, robotic technology introduced demands on staff, disrupted communication due to the reduced ability to maintain visual contact with the full operative field, and increased technical failures [16]. Recently, new multiport robotic surgical systems have increased accessibility and ease of integration into various OR configurations [17]. The Dexter robotic system is an innovative platform with a sterile surgeon's console, two robotic instrument arms, and a robotic endoscope arm. Its compact design allows easy transfer between ORs and integration with existing equipment, enabling quick patient access and seamless switches between laparoscopic and robotic modality. These features make Dexter suitable for teaching programs.

This study evaluated the safety, feasibility, and initial performance of Dexter in cholecystectomy during the learning phase and analyzed its impact on surgeon stress and ergonomics using standardized questionnaires (SMEQ, STAI, and LED) [18, 19, 20].

## 2 Material and Methods

### 2.1 Study Design and Methods

We prospectively collected data from the first 59 patients who underwent RAC with Dexter between December 2022 and June 2024 at Cantonal Hospital Winterthur, Switzerland. Adult patients scheduled for elective LC were included, and informed consent was obtained. The study was approved by the Swiss Ethics Committee (BASEC‐Number 2022‐02169). Three surgeons with varying laparoscopic and robotic experience performed the procedures: two board‐certified surgeons (Surgeon 1 and Surgeon 2) with 7 years of laparoscopic and no prior robotic experience and one (Surgeon 3) with 15 years of robotic experience.

### 2.2 The Dexter Robotic System

Dexter (Distalmotion, Lausanne, Switzerland) features two robotic arms, a camera arm, and a surgeon console. Dexter is an open system, meaning it is compatible with a range of standard OR equipment, including visual imaging systems, electrosurgical units, and commonly used laparoscopic instruments such as trocars, staplers, and advanced energy devices, enabling seamless integration into existing surgical workflows. In our case, we used the existing OR equipment including the TIPCAM1 Rubina 30° 3D endoscopic system (Karl Storz, Tuttlingen, Germany) and the Erbe VIO3 electrosurgical system (Erbe, Tübingen, Germany). Docking is facilitated by incision pointers that align the robotic arms with the trocars. The robotic arms can be folded in a laparoscopic (LAP) position when increased access to the surgical field is needed. When the arms are repositioned into a robotic mode, re‐docking is unnecessary, allowing for a transition that takes only a few seconds. The current portfolio of single‐use instruments includes a needle holder, Johann grasper, Maryland bipolar dissector, monopolar scissors, and monopolar hook. The system provides seven degrees of freedom and a wristed angulation of 75°. The instruments are fully compatible with all 10–12 mm trocars and select 8 mm trocars.

### 2.3 Robotic‐Assisted Cholecystectomy

**FIGURE 1:**
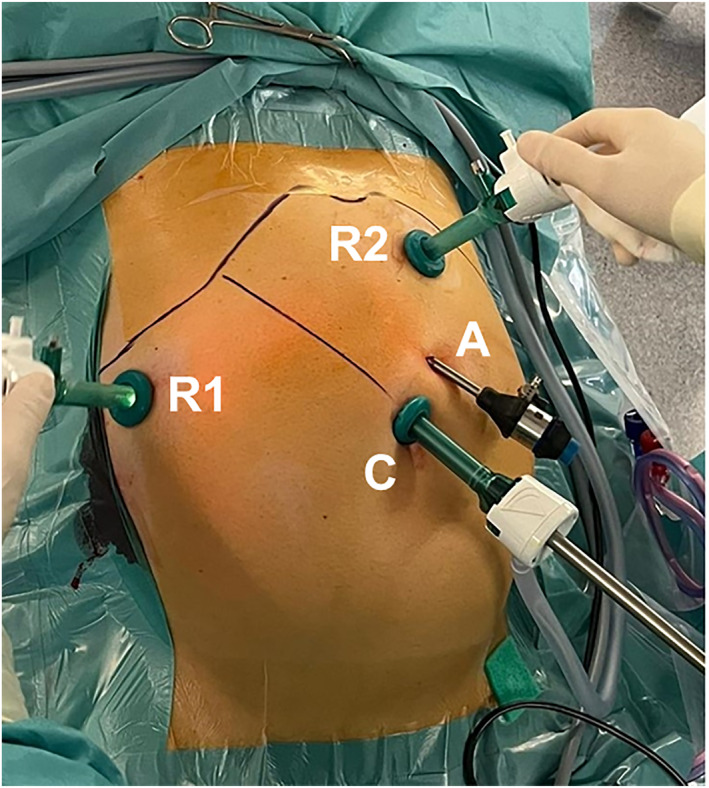
Trocars placement for the cholecystectomy with the Dexter robotic system. R1 and R2 are the right and left robotic trocars arms (11 mm); C is the camera trocar (11 mm) and A is the 5‐mm assistant trocar.

Patients were positioned in the supine position with a 15°–20° Trendelenburg tilt, with legs abducted, and the right arm abducted. The positioning was similar to that used for LC, and the trocar placement followed the laparoscopic approach. An 11‐mm camera port was placed at the umbilicus using the Hasson open technique. Subsequently, two 11 mm trocars for the robotic arms were positioned in the left and right upper abdomen, 14 cm apart. An additional 5‐mm trocar for the assistant port was placed between the umbilicus and the trocar in the left upper abdomen (Figure 1). The docking maneuver was completed, and the robotic system was engaged. The peritoneum over the gallbladder was opened using robotic instruments, exposing the cystic duct and artery. These structures were clipped laparoscopically with Hem‐o‐lok clips (Teleflex Medical, Morrisville, NC, USA) and transected using the same instrument used in LC at our center. The gallbladder was then robotically detached from the liver bed utilizing a Johann grasper and a monopolar hook. At our institution, an assistant aided in stabilizing the gallbladder with a laparoscopic grasper through the assistant trocar. The specimen was extracted with a retriever bag through the umbilical incision. The trocars were removed under direct visualization and the 11 mm fascia was sutured with the help of the Endo Close system (Medtronic, Minneapolis, Mn, USA), using an absorbable suture. The fascia at the umbilical incision was closed with Vicryl (Ethicon Inc, Raritan, Nj, USA).

### 2.4 Data Collection

Demographic data included age of the patient, BMI, ASA score, and indication for the elective cholecystectomy. Perioperative data included total operative time, docking time, console time, intraoperative details, and postoperative complications up to the first follow‐up visit at 30 days. All patients were invited to a standard clinical outpatient follow‐up visit after 30 days; if not possible, telephone consultation was performed.

Docking time was measured from pushing the patient cart toward the operating table until the surgeon was seated at the console. Console time was measured from the first activation of robotic instruments until the surgeon left the console to complete the operation.

The Subjective Mental Effort Questionnaire (SMEQ) is a cognitive workload questionnaire used to measure the perceived mental effort required to perform a task [21]. It consists of a single item where respondents rate their mental efforts on a scale from 0 to 150 points. Nine scale anchors with verbal statements ranging from “no effort at all” to “exceptional amount of effort” are displayed in the SMEQ diagram. Physical discomfort was assessed with the local experience discomfort scale (LED), which allowed surgeons to express their physical discomfort during the surgery. On a scale of 0–10 points, the participants identify their physical discomfort at the neck, trunk, and upper extremities. The total LED score may range from 0 (minimum) to 140 (maximum). For both scales used, a higher score indicates greater mental or physical effort. Figures illustrating the SMEQ and LED questionnaires are described in the studies of van der Schatte Olivier et al. [18] and Grochola et al. [20]. The short, six‐item State‐Trait Anxiety Inventory (STAI) scale was chosen as it is well validated [22, 23]. Each STAI item is scored on a 1–4 likert scale (1, not at all… 4, very much). Total STAI scores range between 6 (minimum) and 24 (maximum), with higher score indicating increased psychological stress. The SMEQ, STAI, and LED questionnaires were completed by the surgeons immediately after each surgery.

### 2.5 Statistical Analysis

Descriptive statistics were used and data were presented as median and interquartile range (IQR). Minitab statistical software was used for statistical evaluation. Significant differences were tested using the Kruskal–Wallis test, with *p* ≤ 0.05 indicating statistical significance.

## 3 Results

Fifty‐nine cholecystectomies were performed between December 2022 and June 2024. The patients' baseline characteristics are shown in Table 1. During the implementation, only elective surgeries were performed robotically with Dexter. All procedures were successfully completed without intraprocedural complications, conversion to open surgery, or major technical failures (Table 2). No additional trocar sites beyond those ones mentioned above were required. In two cases, surgery had to be completed laparoscopically. In one case, a robotic arm collision occurred due to the patient's anatomy, and the inflamed gallbladder was inaccessible robotically. In the second case, a liver tumor was discovered, and the young surgeon was advised to complete the procedure laparoscopically.

**TABLE 1:** Patients baseline characteristics.

Characteristics	*N* = 59
Age (years), median (min–Max)	52 (27–85)
BMI (kg/m^2^), median (min–max)	26.3 (18.3–41.2)
ASA status, *n* (%)	
ASA I	5 (8%)
ASA II	44 (75%)
ASA III	10 (17%)
Indication for surgery, *n* (%)	
Cholecystolithiasis	47 (80%)
Cholecystitis (acute/chronic)	6 (10%)
Choledocholithiasis	1 (2%)
Biliary pancreatitis	3 (5%)
Gallbladder polyps	2 (3%)

**TABLE 2:** Safety variables.

Parameter	Value
Conversion to open, *n* (%)	0 (0)
Intraoperative complications, *n* (%)	0 (0)
Adverse event types, *n* (%)	
Grade 1	4 (6.8%)
Grade 2	0
Grade 3A	1 (1.7%)
Grade 3B	0
Postoperative ERCP, *n* (%)	1 (1.7%)
Length of hospital stay (days), median (min, max)	2 (2–4)

The median skin‐to‐skin operative time was 60 min (IQR 50–78), including a median trocar placement time of 9 min, docking time of 5 min, and console use time of 23 min (IQR 19–34). The median length of stay was 2 days (min, max 2–4).

Postoperatively, five complications occurred during the 30‐day follow‐up period, four of which were minor (C‐D Grade 1), including surgical site infection and postoperative stone passage. One serious complication (C‐D Grade 3A) involved a choledocholithiasis, requiring ERCP to locate and remove bile duct stones.

**FIGURE 2:**
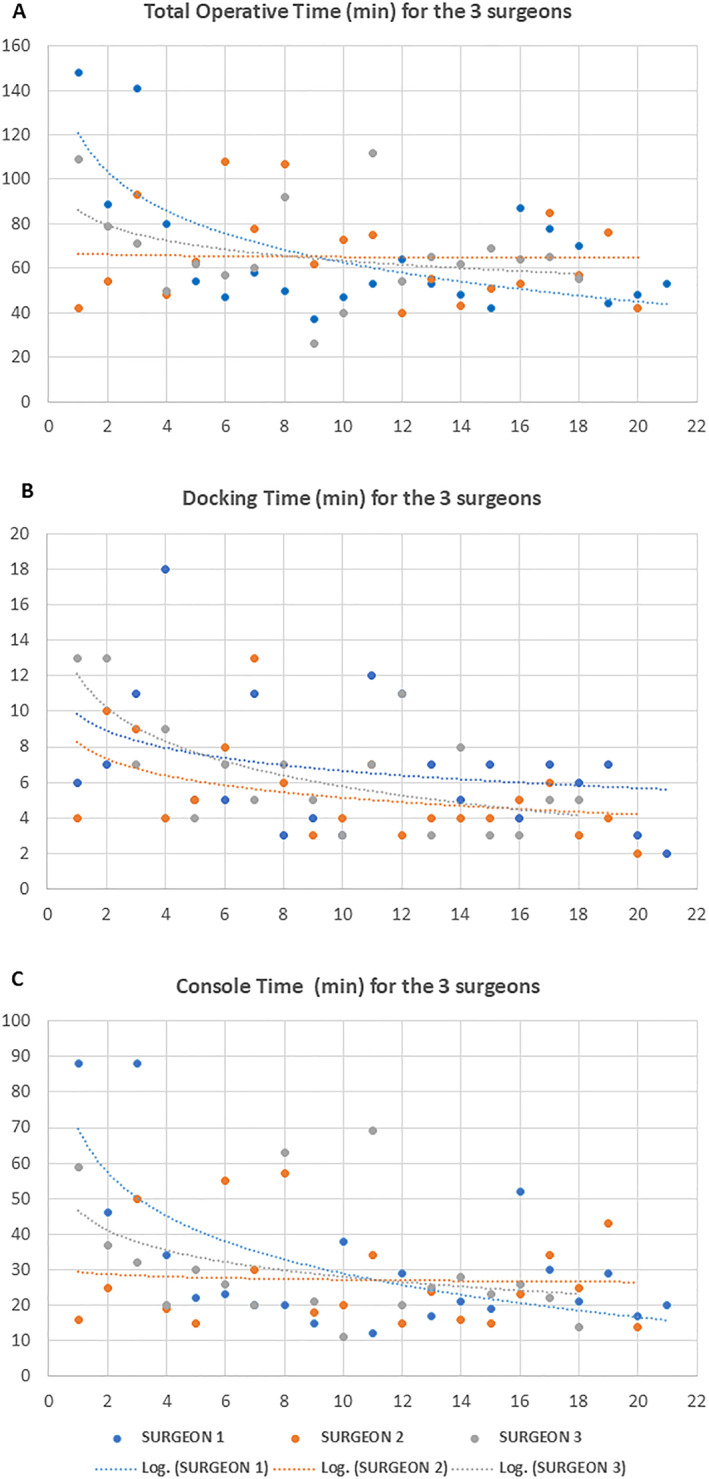
Scatterplot of the (A) operation time (skin‐to‐skin), (B) docking time, and (C) console time of the first consecutive cholecystectomy cases for each surgeon. The time is expressed in minutes. The dotted line represents the logarithmic curve indicating the evolution of the time.

Operating performance by individual surgeons is presented in Table 3. Comparison of total procedure time, docking time, and console time among the three surgeons showed no significant difference. The progression of operative time, docking, and console time per surgeon is shown in Figure 2. After the first 10 cases, both operative and console times improved, with surgeons without prior robotic experience (Surgeons 1 and 2) performing similarly to the experienced robotic surgeon (Surgeon 3). The initial learning curve for docking Dexter was steep and rapid. After fewer than 10 cases, all surgeons achieved similar proficiency, and performance plateaued (Figure 2).

**TABLE 3:** Operative results and surgeon questionnaires results per surgeon.

	Surgeon 1 (*n* = 21)	Surgeon 2 (*n* = 20)	Surgeon 3 (*n* = 18)	*p* value^a^
Total procedure time (min)	53 (48–79)	59 (49–77)	63 (55–73)	*NS*
Trocar placement time (min)	9 (6–10)	9.5 (7.2–13.7)	9.5 (6.7–11)	*NS*
Docking time (min)	6 (4–9)	4 (4–6.7)	6 (3.7–8.2)	*NS*
Console time (min)	22 (19.5–36)	23.5 (16–34)	25.5 (20–33.2)	*NS*
SMEQ questionnaire	15 (10–25)	15 (10–38.7)	10 (0–25)	*NS*
STAI questionnaire	15 (14.8–15)	14.5 (13–15.7)	15 (13.2–15)	*NS*
LED questionnaire	0 (0–1)	7 (6–8.7)	0 (0–4)	*p* = 0.000

*Note:* Data as median and IQR (25th–75th percentile).

^a^ Calculated with the Kruskal–Wallis test.

Surgeon 2 faced unexpected challenges during the learning curve. In one patient, a liver tumor discovery prolonged surgical time to ensure adequate care. Junior laparoscopic surgeons encountered bleeding control challenges in two cases involving the cystic artery. In these inflammatory cases, more delicate Calot's dissection and hemostasis were performed laparoscopically. The flexibility of Dexter allowed rapid conversion to laparoscopy, enabling the surgeries to be completed safely.

The SMEQ questionnaire indicated a low median physical and mental effort (10 points, IQR 10–26.25), with a moderate correlation between the total surgical time and SMEQ values (*r* = 0.574). The stress and anxiety levels, as reported on the STAI questionnaire, were low and comparable among the three surgeons, with a median STAI score of 15 (IQR 13–15), independent of the surgical time (Table 3). The LED scale revealed differences in perceived physical discomfort among the surgeons, but the overall median score was very low (3, IQR 0–6).

## 4 Discussion

Cholecystectomy is one of the most common surgeries performed worldwide and the advancements of robotic‐assisted cholecystectomy have been well documented [6]. Despite criticism of RAC for being costly with uncertain benefits to patients [24], it is frequently used as a “level 1” procedure, particularly for surgeons preparing for more complex robotic operations [4, 7]. Robotic cholecystectomy requires only entry‐level robotic skills. Facilitating access to robotic platforms and accelerating robotic skills training are critical priorities for training the next generation of surgeons and are top concerns for teaching hospitals. However, limited access to a robotic platform for general surgeons remains a significant challenge, hindering broader accessibility. A recent review highlighted potential safety risks when training on cholecystectomy using conventional robotic platforms [24]. Newer, more accessible platforms, such as Dexter, may increase access to robotic‐assisted surgery for a broader range of surgeons while facilitating the safe acquisition of robotic surgery skills. Dexter is an innovative, simple, and highly accessible system that can easily be moved between different ORs. Dexter integrates into the existing laparoscopic workflow; in particular, the trocar configuration used with Dexter closely mirrors a traditional laparoscopic setup. The surgeon remains sterile at the open, draped console, and the robot arms may be folded into a LAP position, providing instant access to the surgical area within seconds. Once the laparoscopic surgical step is completed, the robot arm can be repositioned into a robotic mode without the need to re‐docking. This facilitates a smooth transition to conventional laparoscopy if needed for specific steps of the surgery [25]. These advantages make Dexter particularly well‐suited for integrating robotic surgery training at an early stage of surgical training.

In this study, we describe our initial experience with adopting Dexter in our general surgery department, focusing on the outcomes of the first robotic‐assisted cholecystectomies performed using Dexter. Three surgeons with varying backgrounds and experience in robotic and laparoscopic surgery participated in the study. Our results demonstrate that all surgeons quickly mastered the Dexter system and performed all procedures safely. The learning curves of the surgeons, particularly during docking and console use, revealed that performance converged after 10 cases, indicating a rapid acquisition of robotic skills using Dexter. Previous studies on other multiport robotic platforms have similarly reported rapid learning experiences for robotic‐assisted cholecystectomy, even among surgeons with no prior robotic experience. Oner et al. found that surgeons could achieve proficiency in RAC within 7 to 15 cases, depending on their prior laparoscopic experience [26]. Sasaki et al. [27] also demonstrated that even surgeons without prior robotic training could quickly adapt to a new platform, achieving significant reductions in operative time and complication rates after minimal cases. The operative times observed in our study were comparable to, and in some instance shorter than, those reported for other multiport and modular robotic systems [27, 28, 29]. With Dexter, inexperienced robotic surgeons benefited from the system's flexibility and sterile console option, which allowed a quick switch to laparoscopic surgery if needed. For example, in the event of bleeding from the cystic artery, surgeons could switch to laparoscopic technique to manage the issue, as per their prior training, and then return to the console to complete the procedure. In this setup, our data showed very good safety outcomes with only one serious postoperative complication (Grade 3A): a postoperatively symptomatic choledocholithiasis that required an ERCP. Previous studies suggested that retained or de novo common bile duct stones may occur in 1.8% of patients after laparoscopic cholecystectomy [30] and the ERCP may be required up to 7.4% of patients undergoing robotic cholecystectomy [24]. However, postoperative choledocholithiasis is not directly linked to the specific cholecystectomy technique; rather, it is a general potential complication. The four Clavien–Dindo grade 1 complications were minor; they were detected through close postoperative care and did not delay the postoperative course.

Surgeons typically control the surgical environment through a combination of mental and physical skills, in cooperation with the OR colleagues and available equipment. However, the physical, mental, or emotional strain can reduce an individual's sense of control [18]. Studies have demonstrated that the heart rate variability (HRT), as a measure of total and mental strain, was significantly lower in surgeons performing robotic‐assisted cholecystectomy compared to laparoscopic surgery [31]. These findings suggest that robotic assistance leads to reduced physical and mental strain. Using the NASA Task Load Index, a well‐established tool for assessing mental workload, Stefanidis et al. [32] found that while robotic‐assisted surgery generally imposed lower physical demands compared to traditional laparoscopy, mental workload could be comparable or even higher, depending on the surgeon's experience and the complexity of the procedure. Our SMEQ results indicated that the surgeons experienced relatively low physical and mental effort, with significant variability observed based on prior experience during the early stage of the learning curve. Our STAI findings align with existing research, which generally suggests that while RAS is often associated with lower stress levels due to better control and precision, the learning curve and high expectations associated with robotic systems can still cause some anxiety for some surgeons, particularly in the early stages of adoption [33].

The LED scale in this study showed a low overall discomfort. However, the overall median LED score of 3 is slightly higher than previously reported for robotic surgeries by Grochola et al. and van der Schatte et al. [10, 18]. This could be due to different factors such as the surgeon experience at the time of the study, the innovative robotic system used, the duration of procedures, or individual ergonomic setups [34]. Nonetheless, our findings align with the broader literature, which indicates that robotic surgery typically results in lower physical discomfort compared to traditional laparoscopy, emphasizing the ergonomic benefits of the robotic system even during the learning phase [35, 36].

This study has several limitations to consider. First, the relatively small sample size limits the generalizability of our findings. Establishing a robust CUSUM curve and determining the exact number of cases required to reach proficiency would require a larger cohort of patients per surgeon. Additionally, our study focused on elective surgeries involving simpler cases, as more complex procedures were likely handled by laparoscopic surgeons during this period. Furthermore, patients with potential cancer identified during preoperative evaluations were excluded from robotic surgery with Dexter during the learning phase, potentially biasing our findings toward less challenging cases. Surgeon feedback on using Dexter was collected through subjective questionnaires, which, although validated, are inherently limited by their reliance on personal perception rather than objective data. Another limitation is the lack of a comparative group to serve as a baseline for assessing the surgeons' mental stress, anxiety, or physical discomfort before surgery, limiting our ability to fully interpret the impact of Dexter on surgeons.

Finally, our findings should be validated with larger sample sizes and randomized controlled trials comparing laparoscopic and robotic techniques to better highlight the strengths and capabilities of the Dexter system.

In conclusion, the implementation of the robotic‐assisted surgery with Dexter for simple procedures such as cholecystectomies was safe, feasible, and was easily integrated into routine surgical practice. The system's simplicity and flexibility provided young surgeons with an opportunity to learn robotic surgery techniques, ensuring patient safety and enabling them to quickly perform at a level comparable to experienced robotic surgeons. While the study provides valuable preliminary data, further validation with larger samples and randomized trials is needed to comprehensively evaluate the strengths and capabilities of Dexter in more diverse surgical settings.

## Author Contributions

**Anne‐Sophie Hotz:** writing – original draft. **Nico Seeger:** writing – review and editing. **Lukas Gantner:** validation. **Felix Grieder:** validation. **Stefan Breitenstein:** validation, writing – review and editing.

## Consent

Informed consent was obtained from all individual participants included in the study.

## Conflicts of Interest

N.S., L.G., F.G., and S.B. are consultants for Distalmotion. A.S.H. has no conflict of interest.

## Disclaimer

Dexter Robotic Surgery System, Dexter System, and Dexter are registered trademarks of Distalmotion in the United States and European Union, as well as in certain other jurisdictions.

## Data Availability

The datasets generated during and/or analyzed during the current study are available from the corresponding author on reasonable request.

## References

[1] K. H. Sheetz and J. B. Dimick , “Minimally Invasive Operative Techniques: Is Less Always More?,” Surgery 161, no. 5 (2017): 1455–1457, 10.1016/j.surg.2016.10.037. 27913037

[2] M. A. Abd El Aziz , F. Grass , K. T. Behm , et al., “Trends of Complications and Innovative Techniques’ Utilization for Colectomies in the United States,” Updates in Surgery 73, no. 1 (2021): 101–110, 10.1007/s13304-020-00862-y. 32772277

[3] J. Himpens , G. Leman , and G. B. Cadiere , “Telesurgical Laparoscopic Cholecystectomy,” Surgical Endoscopy 12, no. 8 (1998): 1091, 10.1007/s004649900788. 9685550

[4] J. A. Zaman and T. P. Singh , “The Emerging Role for Robotics in Cholecystectomy: The Dawn of a New Era?,” Hepatobiliary Surgery and Nutrition 7, no. 1 (2018): 21–28, 10.21037/hbsn.2017.03.01. 29531940 PMC5835599

[5] E. Aguayo , V. Dobaria , M. Nakhla , et al., “National Trends and Outcomes of Inpatient Robotic‐Assisted Versus Laparoscopic Cholecystectomy,” Surgery 168, no. 4 (2020): 625–630, 10.1016/j.surg.2020.06.018. 32762874

[6] A. Pordal , J. D. Guerra , D. Morin , W. Oppat , M. J. Jacobs , and S. Patil , “Temporal and Institutional Trends in Robotic Surgery,” Journal of Robotic Surgery 18, no. 1 (2024): 191, 10.1007/s11701-024-01914-w. 38693330

[7] C. M. Haney , E. Karadza , E. F. Limen , et al., “Training and Learning Curves in Minimally Invasive Pancreatic Surgery: From Simulation to Mastery,” Journal of Pancreatology 3, no. 2 (2020): 101–110, 10.1097/jp9.0000000000000050.

[8] A. Farrugia , Q. R. Muhammad , N. T. Ravichandran , M. Ali , G. Marangoni , and J. Ahmad , “Proposed Training Pathway With Initial Experience to Set up Robotic Hepatobiliary and Pancreatic Service,” Journal of Robotic Surgery 16, no. 1 (2022): 65–71, 10.1007/s11701-021-01207-6. 33575862

[9] S. Breitenstein , A. Nocito , M. Puhan , U. Held , M. Weber , and P.‐A. Clavien , “Robotic‐Assisted Versus Laparoscopic Cholecystectomy: Outcome and Cost Analyses of a Case‐Matched Control Study,” Annals of Surgery 247, no. 6 (2008): 987–993, 10.1097/sla.0b013e318172501f. 18520226

[10] L. F. Grochola , C. Soll , A. Zehnder , R. Wyss , P. Herzog , and S. Breitenstein , “Robot‐Assisted Versus Laparoscopic Single‐Incision Cholecystectomy: Results of a Randomized Controlled Trial,” Surgical Endoscopy 33, no. 5 (2019): 1482–1490, 10.1007/s00464-018-6430-7. 30218263

[11] S. Chandhok , P. Chao , J. Koea , and S. Srinivasa , “Robotic‐Assisted Cholecystectomy: Current Status and Future Application,” Laparoscopic, Endoscopic and Robotic Surgery 5, no. 3 (2022): 85–91, 10.1016/j.lers.2022.06.002.

[12] A. Singh , N. S. Panse , V. Prasath , S. Arjani , and R. J. Chokshi , “Cost‐Effectiveness Analysis of Robotic Cholecystectomy in the Treatment of Benign Gallbladder Disease,” Surgery 173, no. 6 (2023): 1323–1328, 10.1016/j.surg.2023.01.017. 36914510

[13] A. P. Ng , Y. Sanaiha , S. S. Bakhtiyar , S. Ebrahimian , C. Branche , and P. Benharash , “National Analysis of Cost Disparities in Robotic‐Assisted Versus Laparoscopic Abdominal Operations,” Surgery 173, no. 6 (2023): 1340–1345, 10.1016/j.surg.2023.02.016. 36959072

[14] T. J. Vidovszky , W. Smith , J. Ghosh , and M. R. Ali , “Robotic Cholecystectomy: Learning Curve, Advantages, and Limitations,” Journal of Surgical Research 136, no. 2 (2006): 172–178, 10.1016/j.jss.2006.03.021. 17059837

[15] F. Kanji , T. Cohen , M. Alfred , et al., “Room Size Influences Flow in Robotic‐Assisted Surgery,” International Journal of Environmental Research and Public Health 18, no. 15 (2021): 7984, 10.3390/ijerph18157984. 34360275 PMC8345669

[16] K. Catchpole , C. Perkins , C. Bresee , et al., “Safety, Efficiency and Learning Curves in Robotic Surgery: A Human Factors Analysis,” Surgical Endoscopy 30, no. 9 (2016): 3749–3761, 10.1007/s00464-015-4671-2. 26675938 PMC13463350

[17] M. Boal , C. G. Di Girasole , F. Tesfai , et al., “Evaluation Status of Current and Emerging Minimally Invasive Robotic Surgical Platforms,” Surgical Endoscopy 38, no. 2 (2024): 554–585, 10.1007/s00464-023-10554-4. 38123746 PMC10830826

[18] R. H. van der Schatte Olivier , C. D. P. van‘t Hullenaar , J. P. Ruurda , and I. A. M. J. Broeders , “Ergonomics, User Comfort, and Performance in Standard and Robot‐Assisted Laparoscopic Surgery,” Surgical Endoscopy 23, no. 6 (2009): 1365–1371, 10.1007/s00464-008-0184-6. 18855053 PMC2687080

[19] S. Arora , T. Tierney , N. Sevdalis , et al., “The Imperial Stress Assessment Tool (ISAT): A Feasible, Reliable and Valid Approach to Measuring Stress in the Operating Room,” World Journal of Surgery 34, no. 8 (2010): 1756–1763, 10.1007/s00268-010-0559-4. 20393847

[20] L. F. Grochola , C. Soll , A. Zehnder , R. Wyss , P. Herzog , and S. Breitenstein , “Robot‐Assisted Single‐Site Compared With Laparoscopic Single‐Incision Cholecystectomy for Benign Gallbladder Disease: Protocol for a Randomized Controlled Trial,” BMC Surgery 17, no. 1 (2017): 13, 10.1186/s12893-017-0206-1. 28183345 PMC5301379

[21] F. R. H. Zijlstra , Efficiency in Work Behaviour: A Design Approach for Modern Tools (Delft, The Netherlands: Delft University Press, 1993).

[22] A. N. Zsido , S. A. Teleki , K. Csokasi , S. Rozsa , and S. A. Bandi , “Development of the Short Version of the Spielberger State—Trait Anxiety Inventory,” Psychiatry Research 291 (2020): 113223, 10.1016/j.psychres.2020.113223. 32563747

[23] T. M. Marteau and H. Bekker , “The Development of a Six‐Item Short‐Form of the State Scale of the Spielberger State—Trait Anxiety Inventory (STAI),” British Journal of Clinical Psychology 31, no. 3 (1992): 301–306, 10.1111/j.2044-8260.1992.tb00997.x. 1393159

[24] S. Kalata , J. R. Thumma , E. C. Norton , J. B. Dimick , and K. H. Sheetz , “Comparative Safety of Robotic‐Assisted vs Laparoscopic Cholecystectomy,” JAMA Surg 158, no. 12 (2023): 1303–1310, 10.1001/jamasurg.2023.4389. 37728932 PMC10512167

[25] D. Hahnloser , D. Rrupa , and F. Grass , “Feasibility of On‐Demand Robotics in Colorectal Surgery: First Cases,” Surgical Endoscopy 37, no. 11 (2023): 8594–8600, 10.1007/s00464-023-10284-7. 37488444 PMC10615910

[26] M. Oner , “Initial Experience of a Single Surgeon for Safety and Feasibility of the Versius Robotic System in Robot‐Assisted Cholecystectomy and Hernia Repair,” Journal of Robotic Surgery 18, no. 1 (2024): 162, 10.1007/s11701-024-01936-4. 38578369

[27] T. Sasaki , F. Tomohisa , M. Nishimura , et al., “Initial 30 Cholecystectomy Procedures Performed With the Senhance Digital Laparoscopy System,” Asian Journal of Endoscopic Surgery 16, no. 2 (2023): 225–232, 10.1111/ases.13143. 36418001

[28] E. Vicente , Y. Quijano , V. Ferri , and R. Caruso , “Robot‐Assisted Cholecystectomy With the New HUGO Robotic‐Assisted System: First Worldwide Report With System Description, Docking Settings, and Video,” Updates in Surgery 75, no. 7 (2023): 2039–2042, 10.1007/s13304-023-01553-0. 37430097

[29] S. Wehrmann , K. Tischendorf , T. Mehlhorn , et al., “Clinical Implementation of the Versius Robotic Surgical System in Visceral Surgery‐A Single Centre Experience and Review of the First 175 Patients,” Surgical Endoscopy 37, no. 1 (2023): 528–534, 10.1007/s00464-022-09526-x. 36002682 PMC9401193

[30] D.‐ho Lee , Y. J. Ahn , H. W. Lee , J. K. Chung , and In M. Jung , “Prevalence and Characteristics of Clinically Significant Retained Common Bile Duct Stones After Laparoscopic Cholecystectomy for Symptomatic Cholelithiasis,” Annals of Surgical Treatment and Research 91, no. 5 (2016): 239, 10.4174/astr.2016.91.5.239. 27847796 PMC5107418

[31] J. Heemskerk , H. R. Zandbergen , S. W. M. Keet , et al., “Relax, Its Just Laparoscopy! A Prospective Randomized Trial on Heart Rate Variability of the Surgeon in Robot‐Assisted Versus Conventional Laparoscopic Cholecystectomy,” Digestive Surgery 31, no. 3 (2014): 225–232, 10.1159/000365580. 25277215

[32] D. Stefanidis , W. W. Hope , and D. J. Scott , “Robotic Suturing on the FLS Model Possesses Construct Validity, is Less Physically Demanding, and is Favored by More Surgeons Compared With Laparoscopy,” Surgical Endoscopy 25, no. 7 (2011): 2141–2146, 10.1007/s00464-010-1512-1. 21184110

[33] O. Lefetz , J.‐M. Baste , J.‐F. Hamel , G. Mordojovich , A. Lefevre‐Scelles , and J.‐M. Coq , “Robotic Surgery and Work‐Related Stress: A Systematic Review,” Applied Ergonomics 117 (2024): 104188, 10.1016/j.apergo.2023.104188. 38301320

[34] L. P. H. Andersen , M. Klein , I. Gögenur , and J. Rosenberg , “Psychological and Physical Stress Among Experienced and Inexperienced Surgeons During Laparoscopic Cholecystectomy,” Surgical Laparoscopy Endoscopy & Percutaneous Techniques 22, no. 1 (2012): 73–78, 10.1097/sle.0b013e3182420acf. 22318065

[35] M. López‐Cano , J. A. Pereira , S. Mojal , et al., “An Ergonomic Study of Single‐Port Versus Multi‐Port Laparoscopic Mesh Insertion for Ventral Hernia Repair,” European Surgical Research 49, no. 3‐4 (2012): 107–112, 10.1159/000342925. 23095250

[36] A. Shugaba , J. E. Lambert , T. M. Bampouras , H. E. Nuttall , C. J. Gaffney , and D. A. Subar , “Should All Minimal Access Surgery Be Robot‐Assisted? A Systematic Review into the Musculoskeletal and Cognitive Demands of Laparoscopic and Robot‐Assisted Laparoscopic Surgery,” Journal of Gastrointestinal Surgery 26, no. 7 (2022): 1520–1530, 10.1007/s11605-022-05319-8. 35426034 PMC9296389

